# 

**Published:** 1921-11

**Authors:** 


### HOSPITAL and HEALTH REVIEW

*Established 1885* No. 1844, Vol. LXXI.

*New Series.* Vol. I. No. 2 *November 1921* Price Sixpence

CONTENTS.

| | PAGE |
|---|---|
| Notes and Comments | 31 |
| The Training of Health Visitors<br>Joseph Cates, M.D., D.P.H. | 35 |
| Quarantine | 36 |
| The late Viscount Sandhurst | 38 |
| Review of the Month<br>Medicine in Arabia. | 39 |
| Difficulties at Burton | 40 |
| Edward Jenner—I.<br>C. J. S. Thompson, M.B.E. | 41 |
| Vaccination and Smallpox | 46 |
| The Purpose of the Hospital<br>The Secretary Superintendent, Middlesex Hospital. | 47 |
| Notable Epidemics. II.—The Sweating Sicknesses. A. T. Nankivell, M.D., D.P.H. | 49 |
| Passing Events and Topics | 51 |
| Reviews of Books | 55 |
| Our Leaflet Competition | 58 |
| Correspondence | 59 |

